# Supplementary material for: Determining the metabolic effects of dietary fat, sugars and fat-sugar interaction using nutritional geometry in a dietary challenge study with male mice
Source: Nat Commun. 2023 Jul 21;14:4409. doi: 10.1038/s41467-023-40039-w (PMC10362033; doi:10.1038/s41467-023-40039-w)
Supplement: Supplementary file 1 — Supplementary Information [file 41467_2023_40039_MOESM1_ESM.pdf]

SUPPLEMENTARY MATERIALS

Title:

Determining the metabolic effects of dietary fat, sugars and fat-sugar interaction using nutritional geometry in a dietary challenge study with male mice

Jibran A. Wali[1,2,*], Duan Ni[1,3], Harrison J.W. Facey[1], Tim Dodgson[1,2], Tamara J. Pulpitel[1,2], Alistair M. Senior[1], David Raubenheimer[1,2], Laurence Macia[1,3,4], Stephen J. Simpson[1,2,*].

1. Charles Perkins Centre, The University of Sydney, Sydney, New South Wales, Australia
2. Faculty of Science, School of Life and Environmental Sciences, The University of Sydney, Sydney, New South Wales, Australia
3. School of Medical Sciences, Chronic Diseases Theme, Faculty of Medicine and Health, The University of Sydney, Sydney, New South Wales, Australia
4. Sydney Cytometry, The University of Sydney, Sydney, New South Wales, Australia

***Corresponding authors**

Emails: jibran.wali@sydney.edu.au ; stephen.simpson@sydney.edu.au

SUPPLEMENTARY INFORMATION

Interpretation of nutritional geometry surfaces

Details for the interpretation of the nutritional geometry (NG) surfaces (e.g., Fig. 1c) have been adapted here and modified from our previous publication [1]. In brief, the phenotype data were analysed by generalized additive modelling (GAM) [2,3] and plotted as response surfaces using thin-plate spline procedures in R software [4], constructed upon nutrient axes for fructose, glucose and fat. These response surfaces map the relationship between metabolic outcomes and the intake of fructose-, glucose- and fat-derived energy (kJ per mouse per d). To aid visualisation, for each response variable, we have presented three 2D sections (slices) through the full, three-dimensional (fructose, glucose, fat) fitted response surface. These three sections were cut through the overall three-nutrient fitted surface at 25% (~5 kJ per mouse per day), 50% (median, ~9 kJ per mouse per day) and 75% (~13 kJ per mouse per day) quantiles of fat intake. These quantiles were calculated from the data for average daily food intake per mouse per cage. The fat intake increases from left to right across the three sections (magnitude is shown in parentheses), while intakes of monomeric fructose and glucose increases along x and y axes within each section, respectively. Across all three sections, dark red indicates the highest value of the phenotypic measurement, while deep blue indicates the lowest value. Phenotypic values remain constant along the black contour lines on the slices, and the numbers along these lines represent the magnitude of the measured parameter. Total energy intake is constant (isocaloric) along any diagonal line with a slope of −1 connecting identical values on x and y axes (for example, purple line in Fig. 1c). For Fig. 1c-d, the purple line is an ‘isocaloric line’. Along the length of this line, the total energy intake remains constant, but the ratio of fructose–glucose eaten changes. The brown line is a ‘food rail’. The ratio of fructose–glucose eaten remains constant along this line, but the total energy intake increases as the line travels away from the origin. The amount of protein (20% of total energy) and starch (30% of carbohydrate energy) in all the diets was fixed; therefore, the energy intake from protein and starch increases only slightly along the food rail vector (for example, the brown line in Fig. 1d) in direct proportion to total energy intake and remains constant along the isocaloric line (purple line in Fig. 1d). When the black lines on the surfaces become parallel (or almost parallel) with the slope of approximately −1 (e.g., as in Fig. S1f), this indicates that the measured parameter increases with total energy intake regardless of the nutrient providing that energy.

Statistical information (obtained from the GAM analysis) for the effects of nutrients and their interaction on the metabolic outcome is provided in the Supplementary Data. For data with statistically significant P values from the GAM analysis ($P < 0.05$), the impact of intake of individual nutrients as well as their combinations on phenotypic parameters can be interpreted from the colours on the NG surfaces.

Study design and animal numbers

The data shown in this study was obtained from a total of 245 mice that were separated into 5 batches or cohorts for logistical reasons (efficient data collection, for performing *in vivo* procedures and for tissue harvesting). For the NG surfaces, data is shown for a maximum of 193 mice that were fed one of 15 isocaloric diets. For 11/15 diets, there were 12 mice/diet giving a total of 132 mice. For one of the diets, 3/12 mice were euthanised due to fight wounds as per ethical guidelines. Therefore, an extra cage of 4 mice was added to make up for the lost mice that led to 13 mice for that diet. For 3/15 diets, an additional 4 mice/diet were run when comparing soy-based diets with the lard-based diets yielding 16 mice/diet. This gives a total of 193 mice maintained on 15 diets and used to plot NG surfaces [(11x12) + 13 + (3x16) = 193].

For the 3 lard-based diets, 12 mice/diet were initially run contemporaneously with their corresponding soy-based diets. For the lard-based diet containing 100% fructose, we had to exclude data for 8/12 mice because of fighting, bullying, aggressive behaviour, and episodes of weight loss. Therefore, we repeated the experiments for this diet by running additional 12 mice and we contemporaneously ran another 12 mice for the 100% fructose-containing soy-based diet. Overall, we used a total of 12 mice/diet for the lard-based diets containing either 100% glucose or 50/50 glucose and fructose. The corresponding soy-based diets had data from a total of 16 mice/diet. For the 100% fructose-containing lard-based diet, data was available for 16 mice (4 mice from the initial experiment and 12 mice from the repeated experiment). For the corresponding 100% fructose-containing soy-based diet, data was available from 28 mice in total (16 mice from the initial experiment and 12 mice from the repeated experiment). After adding the mice from the lard-based diets, the total number of mice used in this study becomes 245 [(193 mice from soy-based diets) + (40 mice from lard-based diets (12+12+16=40)) + (12 extra mice from the 100% fructose-containing soy-based diet)].

Supplementary Fig. S1.

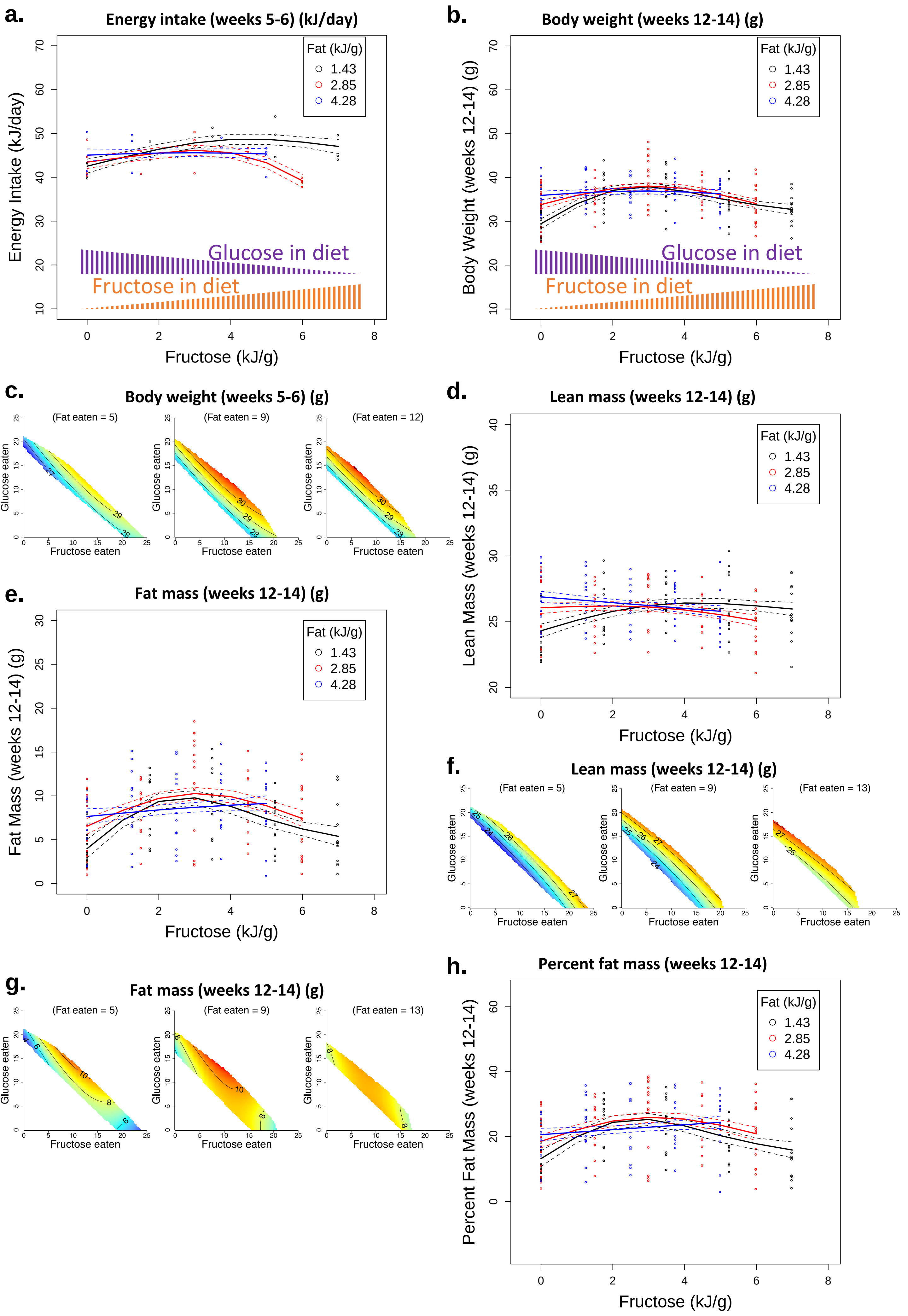

Supplementary Fig. S1. | Related to Fig. 1. (See Supplementary Data 5 for statistics). Source data are provided as a Source Data file.

a. Plot showing the effect of dietary fructose (kJ per g food) on **energy intake** (kJ per mouse per day) at low (10% energy), medium (20%) and high (30%) fat content at 5-6 weeks. Along the x-axis, as fructose levels increase, glucose content in the diet decreases. For diets with a 50:50 fructose:glucose ratio, each monosaccharide was supplied at 3.5, 3.0, and 2.5 kJ per g for the 10%, 20% and 30% fat diets, respectively. Each symbol (o) represents the average energy intake per mouse per cage (n=4 mice per cage). The fitted lines were derived from generalized additive modelling (GAM), fitting an interaction between a smooth term for fructose content (in one dimension) and fat content as a three-level categorical factor, and the dotted lines represent s.e.m. for fitted values.

b. Plot showing the effect of dietary fructose (kJ per g food) on **body weight** (g) at low (10% energy), medium (20%) and high (30%) fat content at 12-14 weeks.

c. Response surfaces showing the relationship between the intake of fructose-, glucose- and fat-derived energy (kJ per mouse per day) and **body weight** (g) of mice at weeks 5-6.

d-e. Plots showing the effect of dietary fructose (kJ per g food) on **lean mass** (g) or **fat mass** (g) at low (10% energy), medium (20%) and high (30%) fat content at 12-14 weeks (lean mass is shown in Fig. S1d and fat mass in Fig. S1e).

f-g. Response surfaces showing the relationship between the intake of fructose-, glucose- and fat-derived energy (kJ per mouse per day) and **lean mass** (g) or **fat mass** (g) of mice at weeks 12-14 (lean mass is shown in Fig. S1f and fat mass in Fig. S1g).

h. Plot showing the effect of dietary fructose (kJ per g food) on **percent fat mass** at low (10% energy), medium (20%) and high (30%) fat content at 12-14 weeks.

Supplementary Fig. S2.

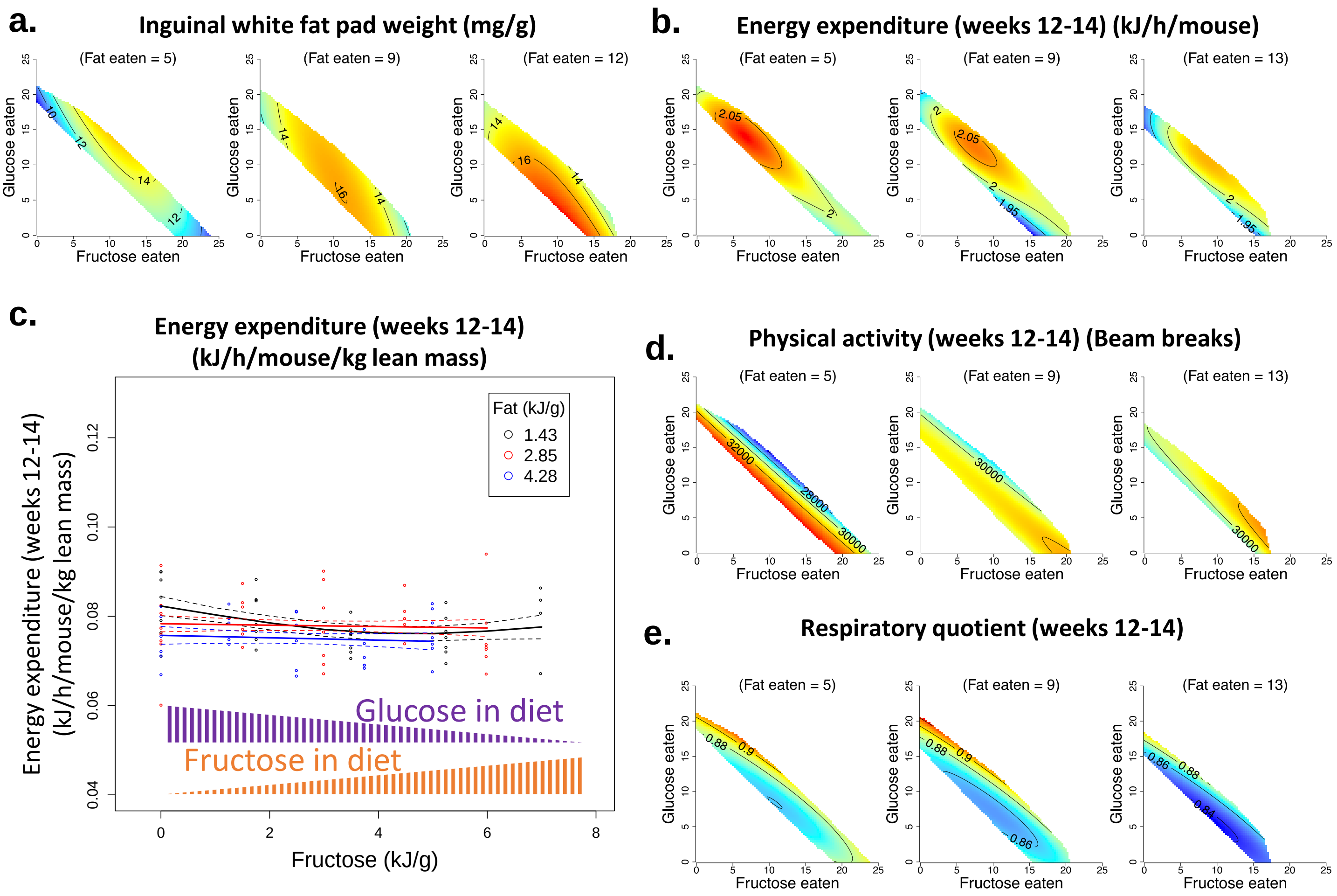

Supplementary Fig. S2. | Related to Fig. 1. (See Supplementary Data 6 for statistics). Source data are provided as a Source Data file.

a. Response surfaces showing the relationship between the intake of fructose-, glucose- and fat-derived energy (kJ per mouse per day) and **inguinal** fat pad weight (mg/g of body weight) of mice at weeks 18-19.

b. Response surfaces showing the relationship between the intake of fructose-, glucose- and fat-derived energy (kJ per mouse per day) and average **energy expenditure** over 24 hours (kJ per hour per mouse) at weeks 12-14.

c. Plot showing the effect of dietary fructose (kJ per g food) on average **energy expenditure** over 24 hours (kJ per hour per mouse per kg lean mass) at weeks 12-14.

d-e. Response surfaces showing the relationship between the intake of fructose-, glucose- and fat-derived energy (kJ per mouse per day) and **physical activity** (beam breaks) (**d**) and average **respiratory quotient** over 24 hours (ratio of carbon dioxide produced and oxygen consumed (**e**) at weeks 12-14.

Supplementary Fig. S3.

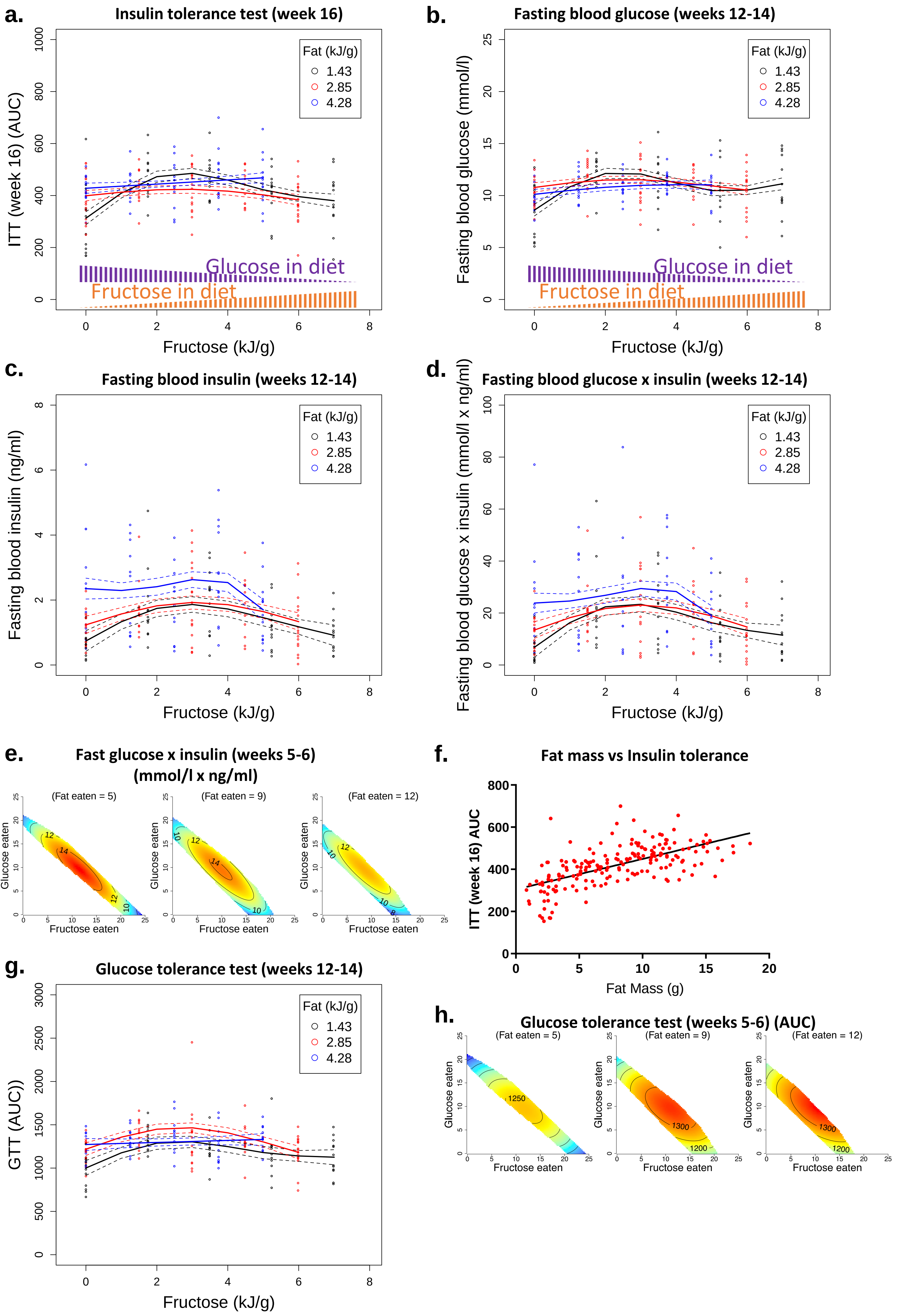

Supplementary Fig. S3. | Related to Fig. 2. (See Supplementary Data 7 for statistics). Source data are provided as a Source Data file.

a-d. Plots showing the effect of dietary fructose (kJ per g food) on **insulin tolerance** (AUC) (**a**) of mice at week 16, **fasting blood glucose concentration** (mmol/l) (**b**), **fasting blood insulin concentration** (ng/ml) (**c**) and their **product** (mmol/l × ng/ml) (**d**) at weeks 12-14.

e. Response surfaces showing the relationship between the intake of fructose-, glucose- and fat-derived energy (kJ per mouse per day) and the **product of fasting blood glucose and fasting blood insulin concentrations** (mmol/l × ng/ml) at weeks 5-6.

f. Relationship between **fat mass** (g) and **insulin tolerance** (AUC) at weeks 12-14 (n=193 mice). R^2 and p value (for the slope of linear regression) for linear regression of data were (R^2=0.4058) and (P=5.723×10^{-22}).

g. Plot showing the effect of dietary fructose (kJ per g food) on **glucose tolerance** (AUC) at low (10% energy), medium (20%) and high (30%) fat content at 12-14 weeks.

h. Response surfaces showing the relationship between the intake of fructose-, glucose- and fat-derived energy (kJ per mouse per day) and glucose tolerance (AUC) at weeks 5-6.

Supplementary Fig. S4.

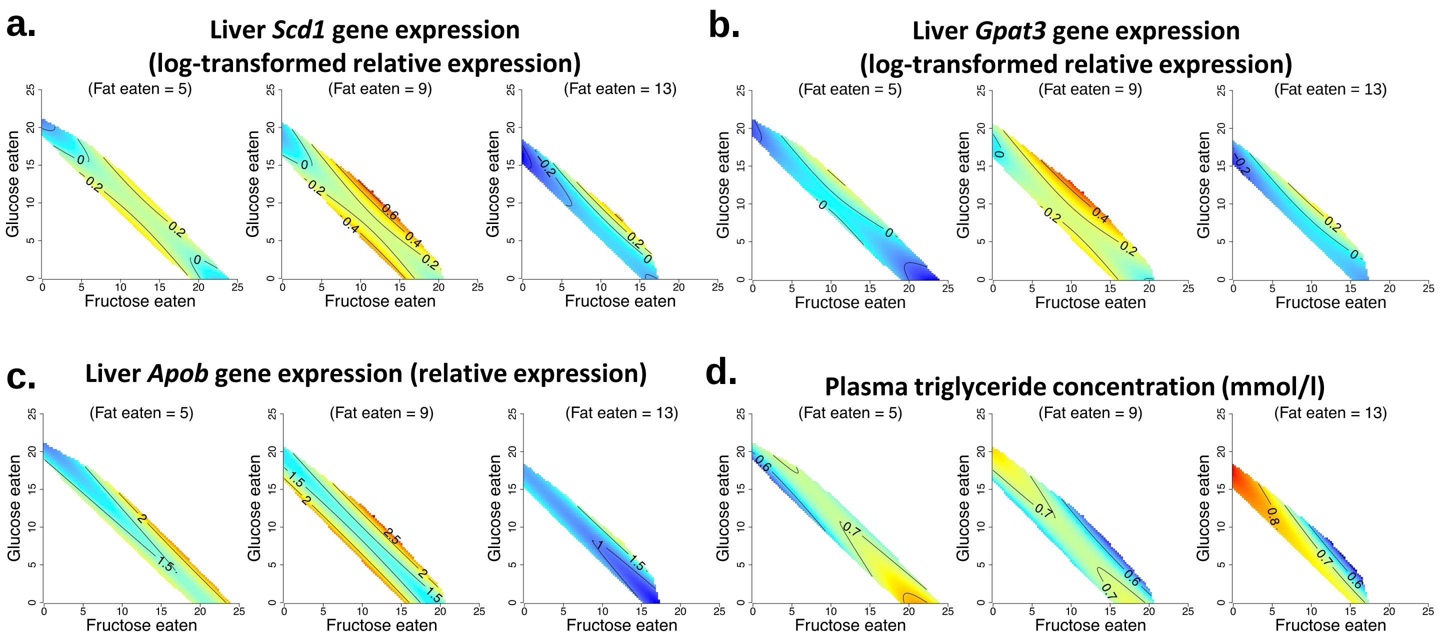

Supplementary Fig. S4. | Related to Fig. 3. (See Supplementary Data 8 for statistics). Source data are provided as a Source Data file.

a-c. Response surfaces showing the relationship between the intake of fructose-, glucose- and fat-derived energy (kJ per mouse per day) and a *de novo* lipogenesis pathway **gene** *Scd1* (**a**), a glycerol synthesis pathway gene, *Gpat3* (**b**), and a lipoprotein transport pathway gene *Apob* (**c**) in livers at weeks 18-19.

d. Response surfaces showing the relationship between the intake of fructose-, glucose- and fat-derived energy (kJ per mouse per day) and **plasma triglyceride** (mmol/l) concentration at weeks 18-19.

Supplementary Fig. S5.

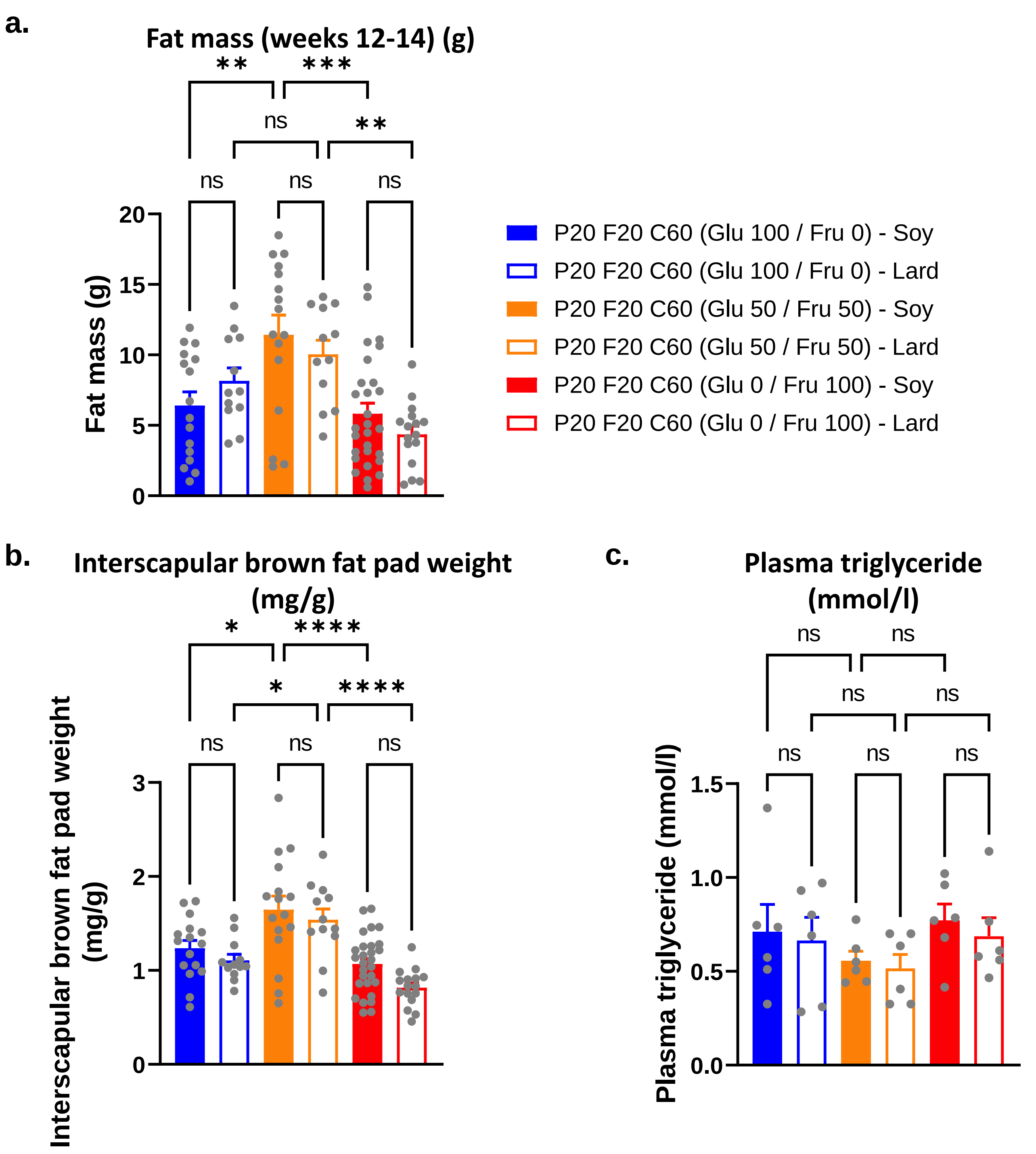

Supplementary Fig. S5. | Related to Fig. 4 & 5. Source data are provided as a Source Data file.

a. Fat mass of mice fed on experimental diets at weeks 12-14. The numbers of animals for G100S, G100L, F50G50S, F50G50L, F100S and F100L were 16, 12, 16, 12, 28 and 16 respectively. ns= not significant, ** P<0.005, *** P<0.001 (P=0.0054 for G100S versus G50F50S, P=0.0002 for G50F50S versus F100S, P=0.0030 for G50F50L versus F100L) for two-way ANOVA (Tukey-Kramer post-hoc test). Mean + s.e.m. Each symbol (➊) represents an individual mouse.

b. Interscapular brown fat pad weight (mg/g body weight) of mice fed on experimental diets at weeks 18-19. The numbers of animals for G100S, G100L, F50G50S, F50G50L, F100S and F100L were 16, 12, 16, 12, 28 and 16 respectively. ns= not significant, * P<0.05, **** P<0.0001 (P=0.0202 for G100S versus G50F50S, P<0.00001 for G50F50S versus F100S, P=0.0458 for G100L versus G50F50L, P<0.0001 for G50F50L versus F100L) for two-way ANOVA (Tukey-Kramer post-hoc test). Mean + s.e.m. Each symbol (➊) represents an individual mouse.

c. Plasma triglyceride concentration (mmol/l) of mice fed on experimental diets at weeks 18-19. The numbers of animals for G100S, G100L, F50G50S, F50G50L, F100S and F100L (n=6 mice per group). No significant difference was reported from two-way ANOVA (Tukey-Kramer post-hoc test). Mean + s.e.m. Each symbol (➊) represents an individual mouse.

REFERENCES

1. Wali, J.A., *et al.* Impact of dietary carbohydrate type and protein-carbohydrate interaction on metabolic health. *Nat Metab* **3**, 810-828 (2021).
2. Solon-Biet, S.M., *et al.* The ratio of macronutrients, not caloric intake, dictates cardiometabolic health, aging, and longevity in ad libitum-fed mice. *Cell Metab* **19**, 418-430 (2014).
3. Hastie, T. & Tibshirani, R. Generalized additive models for medical research. *Statistical methods in medical research* **4**, 187-196 (1995).
4. Core Team, R. R: A language and environment for statistical computing. Vienna, Austria: R Foundation for Statistical Computing. *Available* (2013).
