# Supplementary material for: Determining the metabolic effects of dietary fat, sugars and fat-sugar interaction using nutritional geometry in a dietary challenge study with male mice
Source: Nat Commun. 2023 Jul 21;14:4409. doi: 10.1038/s41467-023-40039-w (PMC10362033; doi:10.1038/s41467-023-40039-w)
Supplement: Supplementary file 3 — Description of Additional Supplementary Files [file 41467_2023_40039_MOESM3_ESM.pdf]

Description of Additional Supplementary Files

Supplementary Data-1

Title: Composition of experimental diets

Description: Composition of experimental diets

Supplementary Data-1a

Title: Composition of experimental diets by % Net Metabolisable Energy derived from protein, fat, carbohydrate, starch, fructose and glucose.

Description: Protein was sourced from casein, fat from soy oil and native starch from wheat. Diets were kept isocaloric by adjusting their cellulose content. The energy obtained from the absorption of digested food is defined as digestible energy (DE) and that portion of energy retained within the body is defined as the metabolizable energy (ME) of that food (DE minus urinary energy losses). The ME minus heat that is released during digestion, absorption and intermediary metabolism of food or microbial fermentation, is known as net metabolizable energy (NME). (References: Elia et al EJCN 2007; Livesey et al Br J Nutr 2001).

Supplementary Data-1b

Title: Composition of experimental diets by % Net Metabolisable Energy derived from protein, fat, carbohydrate, starch, fructose and glucose.

Description: Protein was sourced from casein, fat from lard and native starch from wheat. Diets were kept isocaloric by adjusting their cellulose content.

Supplementary Data-2

Title: Statistical information Related to Figure 1.

Description: Coefficients of the generalised additive models (GAMs) for the data in Figure 1.

Supplementary Data-3

Title: Statistical information Related to Figure 2.

Description: Coefficients of the GAMs for the data in Figure 2.

Supplementary Data-4

Title: Statistical information Related to Figure 3.

Description: Coefficients of the GAMs for the data in Figure 3.

Supplementary Data-5

Title: Statistical information Related to Figure S1.

Description: Coefficients of the GAMs for the data in Figure S1.

Supplementary Data-6

Title: Statistical information Related to Figure S2.

Description: Coefficients of the GAMs for the data in Figure S2.

Supplementary Data-7

Title: Statistical information Related to Figure S3.

Description: Coefficients of the GAMs for the data in Figure S3.

Supplementary Data-8

Title: Statistical information Related to Figure S4.

Description: Coefficients of the GAMs for the data in Figure S4.
