# Supplementary material for: Determining the metabolic effects of dietary fat, sugars and fat-sugar interaction using nutritional geometry in a dietary challenge study with male mice
Source: Nat Commun. 2023 Jul 21;14:4409. doi: 10.1038/s41467-023-40039-w (PMC10362033; doi:10.1038/s41467-023-40039-w)
Supplement: Supplementary file 5 — Reporting Summary [file 41467_2023_40039_MOESM5_ESM.pdf]

nature portfolio

Corresponding author(s): Jibran A. Wali and Stephen J. Simpson

Last updated by author(s): Jul 7, 2023

Reporting Summary

Nature Portfolio wishes to improve the reproducibility of the work that we publish. This form provides structure for consistency and transparency in reporting. For further information on Nature Portfolio policies, see our Editorial Policies and the Editorial Policy Checklist.

Statistics

For all statistical analyses, confirm that the following items are present in the figure legend, table legend, main text, or Methods section.

n/a	Confirmed	
☐	☒	The exact sample size (*n*) for each experimental group/condition, given as a discrete number and unit of measurement
☐	☒	A statement on whether measurements were taken from distinct samples or whether the same sample was measured repeatedly
☐	☒	The statistical test(s) used AND whether they are one- or two-sided<br>*Only common tests should be described solely by name; describe more complex techniques in the Methods section.*
☐	☒	A description of all covariates tested
☐	☒	A description of any assumptions or corrections, such as tests of normality and adjustment for multiple comparisons
☐	☒	A full description of the statistical parameters including central tendency (e.g. means) or other basic estimates (e.g. regression coefficient) AND variation (e.g. standard deviation) or associated estimates of uncertainty (e.g. confidence intervals)
☐	☒	For null hypothesis testing, the test statistic (e.g. *F*, *t*, *r*) with confidence intervals, effect sizes, degrees of freedom and *P* value noted<br>*Give P values as exact values whenever suitable.*
☒	☐	For Bayesian analysis, information on the choice of priors and Markov chain Monte Carlo settings
☒	☐	For hierarchical and complex designs, identification of the appropriate level for tests and full reporting of outcomes
☐	☒	Estimates of effect sizes (e.g. Cohen's *d*, Pearson's *r*), indicating how they were calculated

Our web collection on statistics for biologists contains articles on many of the points above.

Software and code

Policy information about availability of computer code

Data collection

No software was used to collect data.

Data analysis

The following softwares were used to analyse data for this study:
1. R (R Development Core Team) version 4.1.1
2. Graphpad Prism version 9.4.1
3. CalR web application version 1.2 (https://calrapp.org/)

Custom R scripts used for data analysis in this study were uploaded to GitHub and are available at:
https://github.com/Nidane/Sugar-Fat-Study

For questions regarding the data analysis scripts, please write to Dr Alistair Senior.

For manuscripts utilizing custom algorithms or software that are central to the research but not yet described in published literature, software must be made available to editors and reviewers. We strongly encourage code deposition in a community repository (e.g. GitHub). See the Nature Portfolio guidelines for submitting code & software for further information.

Data

Policy information about availability of data

All manuscripts must include a data availability statement. This statement should provide the following information, where applicable:

- Accession codes, unique identifiers, or web links for publicly available datasets
- A description of any restrictions on data availability
- For clinical datasets or third party data, please ensure that the statement adheres to our policy

The data that support the findings of this study are available from the corresponding authors upon reasonable request. Moreover, all the data is available in the Source Data File. All the materials used in this study were commercially available and details have been provided in the methods section of the manuscript. Correspondence and requests for materials should be addressed to Prof Stephen Simpson and Dr Jibran Wali.

Human research participants

Policy information about studies involving human research participants and Sex and Gender in Research.

Reporting on sex and gender	N/A
Population characteristics	N/A
Recruitment	N/A
Ethics oversight	N/A

Note that full information on the approval of the study protocol must also be provided in the manuscript.

Field-specific reporting

Please select the one below that is the best fit for your research. If you are not sure, read the appropriate sections before making your selection.

[x] Life sciences [ ] Behavioural & social sciences [ ] Ecological, evolutionary & environmental sciences

For a reference copy of the document with all sections, see nature.com/documents/nr-reporting-summary-flat.pdf

Life sciences study design

All studies must disclose on these points even when the disclosure is negative.

Sample size	Samples sizes were determined based on the data from our previous dietary studies in mice (Solon-Biet et al, Cell Metabolism 2014; Solon-Biet et al, Cell Metabolism 2016; Solon-Biet et al, Nature Metabolism 2019; Wali et al, Nature Metabolism 2020). For all the dietary groups, we have used at least n=12 mice/diet. This number of mice provides sufficient statistical power (>80%) to detect differences in various markers of metabolic health. For example, using the data for glucose tolerance test (area under the curve) for two dietary groups with a difference in mean AUC of 150 and standard deviation of 90, a sample size of 12 provides 80% statistical power. Therefore, our sample sizes had sufficient power to detect phenotypic differences between the dietary groups.
Data exclusions	Details of mice euthanised at different time-points before the completion of dietary experiment and the reasons for exclusion are provided in Supplementary Information. Once the lesions or serious fight wounds were noticed, mice were excluded from in vivo procedures and were not included in in vitro experiments. For all the in vivo procedures and in vitro experiments, any mouse/tissue sample with a known error in measurement was excluded from final analyses.
Replication	Attempts at replication were successful. Mice arrived in time-staggered cohorts, with diet treatment spread across cohorts,minimising the bias effect of cohort or batches of mice. Cohort, cage and mice were replicated at each stage. The animals were spread across a total of five cohorts (details in Supplementary Information and Methods section). In vitro analysis of bio-banked tissues were analysed with sample randomisation using biological and technical replicates. A minimum of 3 independent experiments were performed for each in vitro data item.
Randomization	Mice arrived in time-staggered cohorts and were randomly allocated to 4 animals per cage. Diet treatments were staggered within and between cohorts to ensure randomization of cohort and diet effects.<br>For in vitro experiments such as qPCRs, ELISA and histology work, samples from n = 4-6 mice/diet were randomly selected. For these experiments, each ELISA and qPCR plate was set up such that samples from each diet were included in every plate. Thus, samples from any given dietary treatment were not concentrated on a single plate, but were spread across the various plates. This was done to minimize any impact of plate-to-plate variations on the overall outcomes of the experiments. Similarly, for histology work, the specimens were processed in batches such that each diet was represented in each batch of samples.
Blinding	For in vitro experiments such as qPCRs, ELISA and histology work, our aim was to randomly select n = 4-6 mice/diet. To achieve this objective, investigators were required to be aware of the dietary treatment that each mouse received. However. once the mouse samples were selected, all samples were assigned a unique ID number that did not reveal the dietary treatment of the sample and all analyses were

performed using this number. This ensured blinding to dietary treatments when performing experimental work. After performing data analysis, sample results were grouped by dietary treatment to make plots and graphs.
In vivo procedures such as longitudinal food intake measurements required the same diet treatments to be added to cages throughout the study. Therefore, for food intake measurements, investigators were required to know treatment allocations at the cage level. Similarly, after in vivo assessment of glucose tolerance, mice required to be fed again with correct experimental diet and housed in correct cages. Thus, blinding was not feasible for such procedures.

Reporting for specific materials, systems and methods

We require information from authors about some types of materials, experimental systems and methods used in many studies. Here, indicate whether each material, system or method listed is relevant to your study. If you are not sure if a list item applies to your research, read the appropriate section before selecting a response.

Materials & experimental systems

n/a	Involved in the study	
☒	☐	Antibodies
☒	☐	Eukaryotic cell lines
☒	☐	Palaeontology and archaeology
☐	☒	Animals and other organisms
☒	☐	Clinical data
☒	☐	Dual use research of concern

Methods

n/a	Involved in the study	
☒	☐	ChIP-seq
☒	☐	Flow cytometry
☒	☐	MRI-based neuroimaging

Animals and other research organisms

Policy information about studies involving animals; ARRIVE guidelines recommended for reporting animal research, and Sex and Gender in Research

Laboratory animals	C57BL/6J male mice (4 weeks old) were purchased from Animal Resources Centre (Western Australia).
Wild animals	This study did not involve wild animals.
Reporting on sex	Only male mice were used in this study. Male mice were used because males are more prone to diet-induced obesity and insulin resistance than females.
Field-collected samples	This study did not involve samples collected from the field.
Ethics oversight	All animal procedures and protocols were approved by the institutional animal ethics committee at the University of Sydney (Protocol No. 2018/1362).

Note that full information on the approval of the study protocol must also be provided in the manuscript.
